# Investigation of risk of dementia diagnosis and death in patients in older people's secondary care mental health services

**DOI:** 10.1002/gps.5455

**Published:** 2020-11-04

**Authors:** Anne Kershenbaum, Rudolf N. Cardinal, Shanquan Chen, Benjamin R. Underwood, Aida Seyedsalehi, Jonathan Lewis, Judy Sasha Rubinsztein

**Affiliations:** ^1^ Cambridgeshire and Peterborough NHS Foundation Trust University of Cambridge Cambridge UK; ^2^ University of Cambridge Cambridge UK; ^3^ Cambridgeshire and Peterborough NHS Foundation Trust The Gnodde Goldman Sachs Translational Neuroscience Unit University of Cambridge, Cambridge UK; ^4^ Cambridgeshire and Peterborough NHS Foundation Trust Cambridge UK

**Keywords:** anxiety disorders, bipolar disorder, dementia, depression, epidemiology, mortality, older people's mental health services, outcome studies, schizophrenia

## Abstract

**Objectives:**

Previous studies have shown increased rates of death and dementia in older people in specific serious mental illnesses (SMI) such as bipolar disorder or depression. We examined the rates of death and dementia in older people referred into a secondary care psychiatric service across a range of SMIs.

**Methods:**

We used an anonymised dataset across 6 consecutive years with 28,340 patients aged 65 years and older from a single secondary care psychiatric trust in the United Kingdom.

We identified deaths and incident dementia in patients with bipolar disorder/mania, schizophrenia, recurrent depression and anxiety disorders**.** We compared mortality and dementia rates between these diagnostic groups and in different treatment settings. We also examined mortality rates and dementia rates compared with general population rates.

**Results:**

Patients with schizophrenia showed the highest hazard rate for death compared to other groups with SMIs (hazard ratio, 1.58; 95% confidence interval (CI), 1.18–2.1, with anxiety group the reference). Survival was reduced in patients referred to liaison psychiatry services. There were no significant differences between the SMI groups in terms of rates of dementia. However, risks of death and dementia were significantly increased compared to the general population (standardized mortality rates with 95% CI, 2.6(2.0–3.3), 3.5(2.6–4.5), 2.5(2.0–3.0) and 1.8 (1.4–2.2) and standardized dementia incidence rates with 95% CI, 2.7(1.5–4.1), 2.9(1.5–4.7), 3.8(2.6–5.2) and 4.3 (3.0–5.7) for bipolar disorder/mania, schizophrenia, recurrent depression and anxiety disorders respectively.

**Conclusions:**

Older adults referred into an old age psychiatry service show higher rates of dementia and death than those reported for the general population.

## 1 INTRODUCTION

Patients with severe mental illness (SMI) tend on average to die younger than the general population.^1^, ^2^, ^3^, ^4^, ^5^ Though most studies have focused on younger people (<65 years of age), increased mortality in patients with psychiatric disorders has recently been found to extend to older people.^6^ ^,^ ^7^ In addition, an increased risk of dementia has been noted in patients with depression,^8^ bipolar disorder^9^ and psychotic disorders.^10^ ^,^ ^11^ The causes of this increased risk of death and of dementia in patients with psychiatric disorders are not clear but are probably multiple and may differ between different disorders.

**Key points**

- Older adults referred for specialist assessment at an old age psychiatry service showed higher rates of dementia and death than those reported for the general population
- Older patients with serious mental illnesses referred into liaison psychiatry (general hospital) showed increased mortality rates compared to patients referred into other referral routes suggesting that physical illnesses underlie the increased mortality
- Within the serious mental illness groups, schizophrenia showed the highest death rate

In this study, we examine death and dementia rates in a secondary health care setting, across a range of SMIs, namely, bipolar disorder/mania, schizophrenia, recurrent depression and anxiety disorders. Previous studies have examined the risk of death or dementia in specific disorders such as bipolar disorder. Other disorders, for example anxiety disorders, have been much less well explored in terms of their relationship to risk of death or dementia. We also compare our measured rates to general population rates, to test the hypothesis that presentation with these conditions to secondary care services is associated with an increased risk of death and diagnosis.

This is the first time, to our knowledge, that such an examination of the risk of death or dementia in an older people (65 years and older) has been examined using an anonymised dataset of this magnitude (28,340 patients), in a single mental health trust, in the United Kingdom. We have examined data over a 6‐year period. A previous large‐scale record‐based study examining risk of death and dementia in bipolar disorder in Western Australia, examined only men,^6^ but we have included both men and women in our study. Additionally, we have examined the effect of treatment setting to identify the most at‐risk population within our service.

## 2 MATERIALS AND METHODS

Using the electronic patient record, we identified a retrospective cohort of older patients who had been referred to Cambridge and Peterborough NHS Foundation Trust (CPFT) mental health services. CPFT provides mental health services to Cambridgeshire, serving a population of approximately 1 million people, of which 165,000 were over the age of 65 at the last census in 2016. Older patients were referred into CPFT services to a variety of treatment settings (as detailed below). The CPFT Research database (NHS Research Ethics Reference 17/EE/0442) stores electronic record data in a de‐identified form generated using CRATE (Clinical Records Anonymisation and Text Extraction) software^12^ which de‐identifies the clinical record and provides an arbitrary patient‐specific identifier. Referral data to CPFT, along with demographic, admission and diagnosis variables have been collected electronically by the current data collection system (Servelec RiO electronic care record system) since 2012, fully operational since 2013. The start of study was taken as 01 January 2013 and the end of study 19 August 2019 (referral records were available until 19 August 2019). The study had Health Research Authority approval (IRAS number 241365).

After exclusion of referral records before 2013, we extracted the first referral record per patient (index referral; Figure S1). We merged demographic variables (date of birth, sex and date of death) with the referral data. Patients aged 65 or older at first referral were extracted to create the population of patients referred to CPFT mental health services aged 65 or older since 2013 (base population). We linked coded International Classification of Disease‐10 (ICD‐10) diagnoses to the referral and demographic data.

Treatment setting was defined as the service the patient was first seen by following the index referral. A patient may be referred into services again after the index referral during the follow‐up period, but the treatment setting associated with the index referral was the one recorded. We derived treatment setting from a referral variable, ‘team referred to’. This referral variable included liaison psychiatry (patients admitted to general acute hospitals), memory services, crisis and home treatment teams, and others, which was the largest group and received referrals from primary care mental health service, community older people's teams, psychotherapy services and all other referral routes. There were thus four main four treatment setting groups for older people.

Referral, diagnosis and demographic tables were extracted on 11 April 2020.

Structured Query Language queries were used to extract data from the CPFT Research database and LibreOffice Calc for spreadsheet functions. R was used (version 3.6.2) for data manipulation and statistical analysis^13^; package lubridate was version 1.7.4.

### 2.1 Identification of bipolar disorder or mania/schizophrenia/recurrent depression/anxiety (four SMI groups)

Seventeen thousand six hundred and sixty‐two of the 28,340 (62.3%) people in the base population had a diagnosis code assigned to their record (Table S1). The diagnoses are clinician‐entered.

We extracted ICD‐10 codes F31* (bipolar affective disorder) and F30* (mania), identifying 401 patients with a bipolar disorder/mania diagnosis. We also identified patients with a diagnosis of schizophrenia, F20* (290 patients); recurrent depressive disorders, F33* (849 patients) and anxiety disorders, F40* and F41* (1152 patients). These four SMI groups were chosen to represent four common chronic psychiatric disorders within which to measure the rate of outcomes, death and dementia. We used a hierarchical approach to group patients. All patients with a bipolar disorder/mania code (F31*/F30*) are included into the bipolar disorder/mania group; patients with a schizophrenia code (F20*) are in the schizophrenia group if not already in the bipolar disorder/mania group; patients with a recurrent depression code (F33*) fall into the recurrent depression group if not already in the bipolar/mania or schizophrenia group and patients with an anxiety code (F40*–41*) are in the anxiety group if not already in the other groups.

Additionally, for a sensitivity analysis, a variable was created indicating SMI group without using a hierarchical approach but allowing separate groupings for all possible SMI combinations (where SMIs were not mutually exclusive).

### 2.2 Identification of death (outcome)

Dates of death were available in the de‐identified electronic records, through central NHS reporting to Trusts.

### 2.3 Identification of dementia diagnosis (outcome)

Diagnoses of dementia (F00*–F03*) had similarly been entered by clinicians, with an associated date which represents either the onset of the dementia or the date of entry (dementia date). Patients were excluded who had dementia coded before, or on the same day as, the SMI (bipolar disorder/mania, schizophrenia, recurrent depression and anxiety disorders).Whereas death was automatically recorded even for patients discharged from CPFT, dementia was only coded in our records while the patient was under CPFT's care. Patients could be discharged and then re‐referred. If re‐referred back in with a new dementia, we would expect this new dementia diagnosis to be coded after re‐referral. We therefore took each patient's ‘end’ date as the last date of discharge from CPFT services, or the end of the study if they had not been discharged by then.

### 2.4 Analysis

The start of follow‐up for both death and dementia risk analysis, was the date associated with the SMI diagnosis code or a year after referral, whichever was the latest.

To examine the risk of death, patients were followed up until death or the end of the study, whichever came first. To examine the risk of dementia, patients were followed up until a diagnosis of dementia, and if no dementia, until death, or the last date of discharge from CPFT, whichever came first.

**FIGURE 1:**
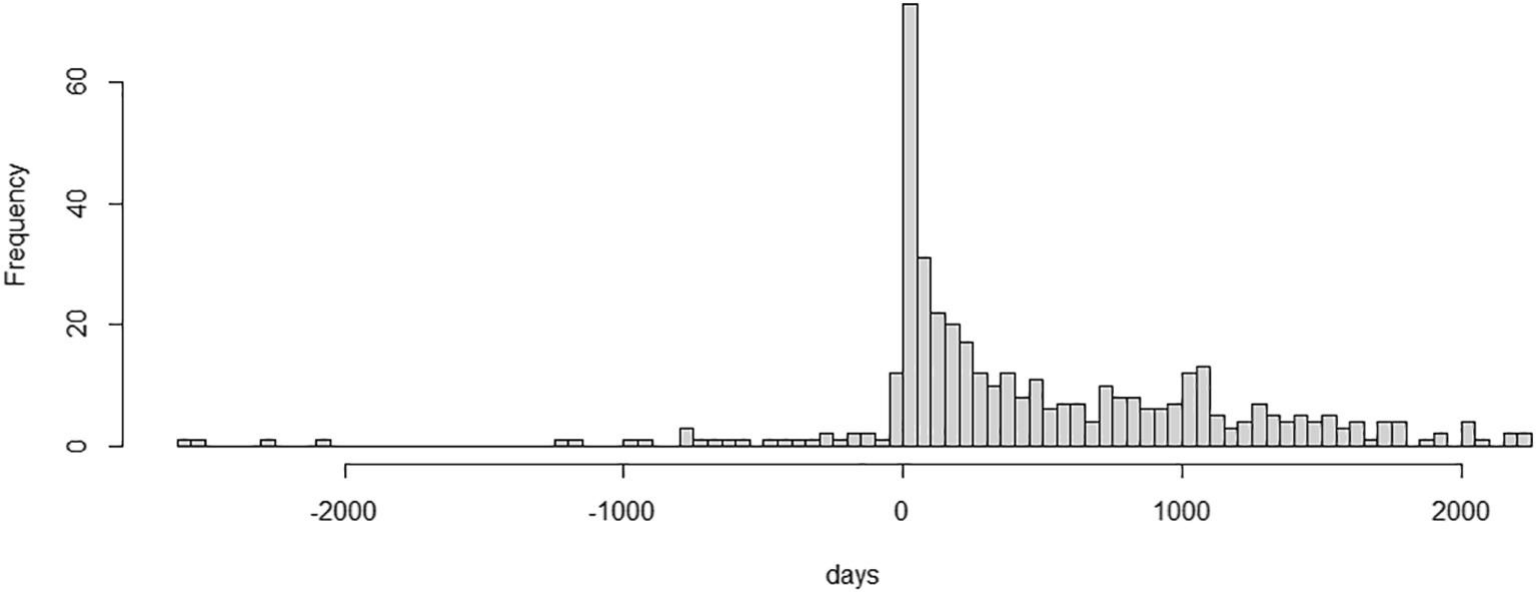
Histogram of dementia diagnoses (*N* = 419) in relation to referral date showing excess of diagnosis entry just after referral in four serious mental illness groups, *N* = 2572. Follow‐up starting from at least a year after referral was applied in analysis. This allowed exclusion of prevalent cases of dementia at referral from the cohorts, allowing a year for entry of the dementia code

Follow‐up starting from at least a year after referral was applied to both death and dementia analysis. This allowed exclusion of prevalent cases of dementia at referral from the cohorts, allowing a year for entry of the dementia code. The histogram of dementia diagnoses in relation to referral date (Figure 1) showed the expected excess of diagnoses in the year after referral, which subsequently levelled out. For mortality analysis, starting follow‐up from at least a year after referral, allowed time for diagnoses to be entered before death. Patients with the dementia date or death date on the same day or before the SMI diagnosis code were also excluded.

After exclusion of those with less than a year of follow‐up time after referral, 2186 out of the 2572 in the four SMI groups remained for follow‐up for death and 1403 for dementia. Then after exclusion of those with the death date before the SMI code, 2185 remained for follow‐up for death, and after exclusion of those with the dementia date on the same day or before the SMI code, 1339 remained for follow‐up for dementia.

#### 2.4.1 Death rates

We used Kaplan–Meier survival analysis to examine time to death from referral comparing the four SMI groups. We explored death rates, examining age, SMI exposure, gender and treatment setting in a univariate analysis. We compared survival in different strata using R functions survfit and ggsurvplot which uses the log‐rank test to compare strata. We explored death rates in Cox analysis in the four SMI groups, controlling for age, gender and treatment setting. We performed three Cox sensitivity analysis. (A) First, we repeated the analysis in the four SMI groups, controlling for age, gender and treatment setting as before, but now also controlling for dementia. (B) Second, we repeated the analysis after removal of all patients with a diagnosis of dementia. (C) Finally, we repeated the analysis in SMI groups that were not mutually exclusive, again controlling for age, gender and treatment setting.

We calculated the standardised mortality ratio (SMR), the ratio of observed to expected deaths in the four SMI groups in age groups, for males and females separately. We measured the follow‐up time of study participants (in days) in 5‐year age groups and with males and females separately, where one individual can contribute follow‐up time to more than one 5‐year age group. We recorded the observed number of deaths in 5‐year age groups with males and females separately in each SMI group, using the death date to identify the appropriate age group. We estimated the expected number of deaths in the general population using mortality rates from the Office for National Statistics death registrations for England and Wales, 2016,^14^ given the same follow‐up time. General population death rates, given as registered deaths in a calendar year, were converted for use in the study to deaths per day of follow‐up. For example, in females age 65–69, the general population figure of 9.6/1000 per year was taken as 9.6 deaths per 365,000 follow‐up days for use in the study. We then calculated the expected deaths in the general population given the follow‐up time in the study population of females ages 65–69 of a given SMI exposure. We measured the ratio of observed to expected deaths to derive the SMR in the subgroup. We calculated the SMR for each SMI, by summing the observed deaths and the expected deaths as calculated above in the age and gender subgroups and taking the ratio. We calculated upper and lower 95% confidence limits for the SMR values using the Vandenbroucke method.^15^ In a sensitivity analysis, we repeated the calculation of SMR for each SMI after removal of all patients with a diagnosis of dementia, using the same cases as used in Cox sensitivity analysis (B).

#### 2.4.2 Risk of dementia diagnosis

We used competing risk analysis to examine the risk of dementia over time in the context of a competing outcome of death, comparing the four SMI groups. In a sensitivity analysis, we repeated this competing risk analysis in SMI groups which were not mutually exclusive. Competing risk analysis^16^ was used because in examination of the outcome of interest, dementia, death is a competing outcome that precludes the subsequent development of dementia.

We calculated the standardised incidence ratio (SIR), the ratio of observed to expected dementia cases in the four SMI groups in age groups, as described above in calculation of SMR (Section 2.4.1). Here we estimated the expected number of new cases of dementia in the general population using incidence by age group in the UK^17^ given the same follow‐up time.

## 3 RESULTS

### 3.1 Descriptive statistics

In the study population of four SMI groups (N = 2572), the proportions of patients in the younger age categories (65–74) were lower in the recurrent depression and anxiety groups than in the schizophrenia and bipolar groups (Table S2).

### 3.2 Mortality

After exclusions, there were 2185 patients for mortality analysis. Within the 2185, there were 80/355 (22.5%) patients in the bipolar disorder/mania group with death recorded, 65/238 (27.3%) in the schizophrenia group, 184/715 (25.7%) in the recurrent depression group and 163/877 (18.6%) in the anxiety group.

Kaplan–Meier analysis showed a significant difference (*p* = 0.038) in survival between the four SMI groups (Figure 2), and a significant difference (*p* < 0.0001) in survival between treatment setting. In the Cox analysis controlling for age at referral (age rather than age group), treatment setting and gender, schizophrenia showed the lowest survival, (hazard ratio (HR) 1.58, *p* = 0.002, with anxiety group the reference). Recurrent depression also showed a significantly lower survival compared to anxiety, (HR 1.29, *p* = 0.018). Survival was lower in males, older people and those referred into liaison psychiatry (respectively, HR 1.57, *p* < 0.001; HR 1.09, *p* < 0.001, for each year and HR 1.88, *p* < 0.001, with general psychiatry the reference). In Cox sensitivity analysis (A) controlling for age gender, and treatment setting as before, but now also controlling for dementia, survival was again lower in males, older people, those referred into liaison psychiatry compared to general psychiatry and in schizophrenia (HR 1.56, *p* = 0.003) and recurrent depression (HR 1.26, *p* = 0.034) compared to anxiety. Those with dementia showed a significantly lower survival than those without (HR 1.50, *p* < 0.001; Figure S2a). In Cox sensitivity analysis (B), excluding patients with dementia, survival was again lower in males, older people, those referred into liaison psychiatry compared to general psychiatry and in schizophrenia compared to anxiety. However, in this analysis, survival in recurrent depression was not significantly different from anxiety (HR 1.22, *p* = 0.119; Figure S2b), though we note the effect size was only marginally lower than in the main analysis (HR 1.29 as above). In Cox sensitivity analysis (C), in SMI groups which were not mutually exclusive, again controlling for age, gender and treatment setting, survival was lower in schizophrenia only and recurrent depression only groups compared to anxiety only. Also, the recurrent depression plus schizophrenia group showed particularly lower survival compared to the anxiety only group (HR 11.7, *p* < 0.001; Table S3). SMRs, observed deaths as a ratio to expected in the general population given the same follow‐up time, were greater than one in 31/32 subgroups (in one subgroup no death was recorded), although some comparisons did not reach statistical significance (Table 1). SMRs were highest in the younger age categories (65–69 and 70–74) in all SMI groups across males and females, except in males with schizophrenia. The SMR for schizophrenia (all eight subgroups combined) was the highest of the four SMI groups (3.5 [95% confidence interval (CI) 2.6–4.5]), and for anxiety the lowest (1.8 [95% CI 1.4–2.2]). In the sensitivity analysis excluding patients with dementia, the same general pattern of results was found, with schizophrenia showing the highest SMR (3.3 [95% CI 2.4–4.4]) and anxiety the lowest (1.7 [95% CI 1.4–2.2]; Table S4). SMRs for each SMI were slightly lower than found in the main analysis.

FIGURE 2Survival, by serious mental illness (SMI) and initial treatment setting, in patients with at least a year of follow‐up (2185 people). (A) Patients with schizophrenia and recurrent depression had higher mortality than the anxiety group. (B) Patients referred to liaison psychiatry services had higher mortality than those referred to memory services/crisis resolution or home treatment teams/other treatment settings. (C) Hazard ratios increased by age, higher in males versus females, highest in liaison in comparison between treatment settings and highest in schizophrenia in comparison between SMIs

**Figure:**
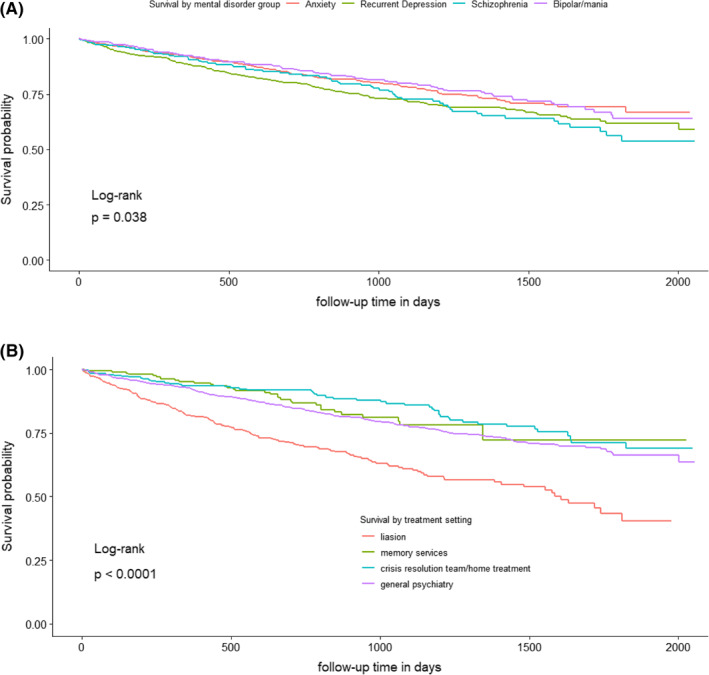

**Figure:**
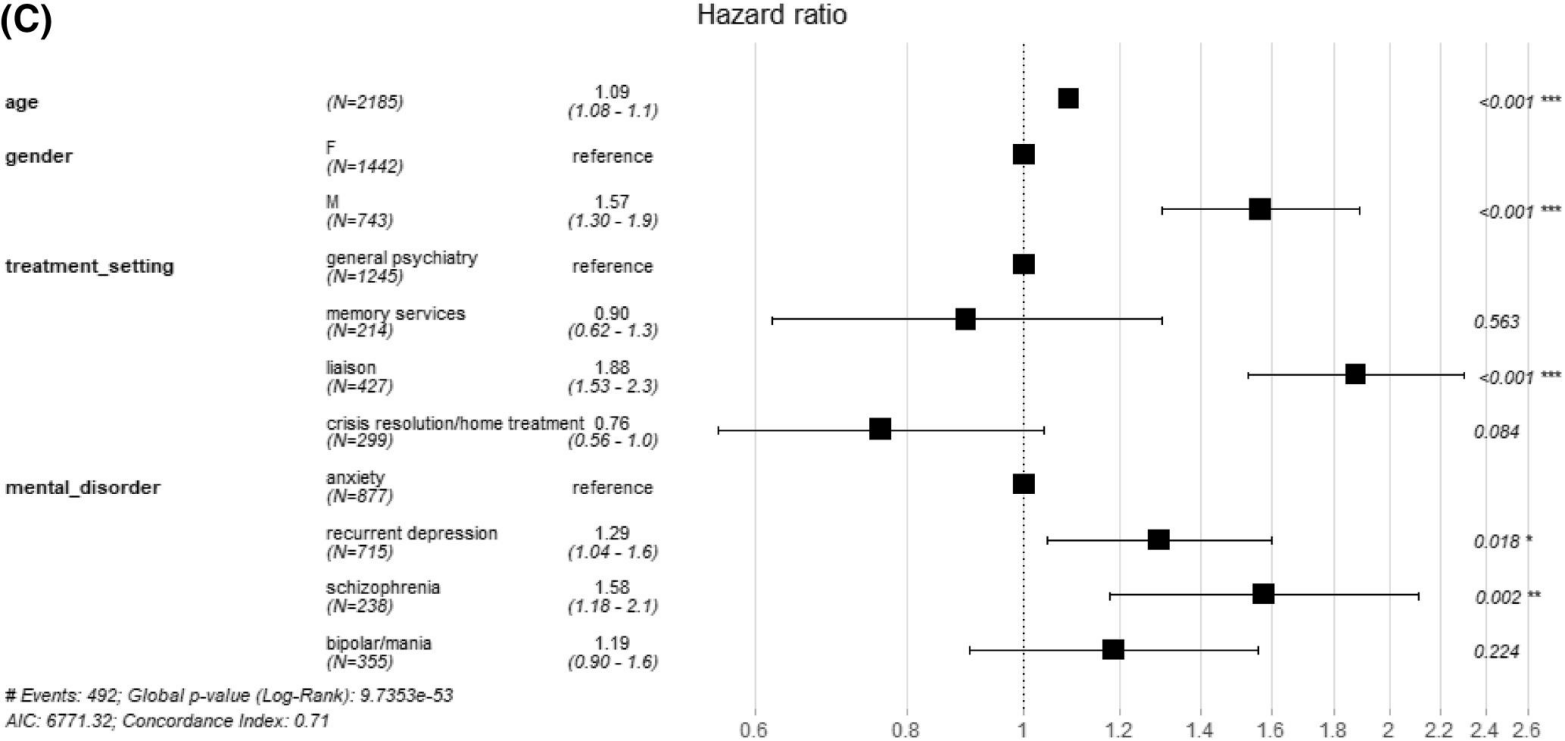

**TABLE 1:** Observed deaths and dementia cases in study (O) and expected in general population given same follow‐up time (E) in patients with at least a year of follow‐up from referral

			Deaths					Dementia incident cases				
SMI		Age	*N*	O	E	SMR (95% CI) for subgroup	SMR (95% CI) for disorder	*N*	O	E	SIR (95% CI) for subgroup	SIR (95% CI) for disorder
Bipolar/mania	F	65–69	192	5	0.9	**5.4 (1.7**–**11.2)**	**2.6 (2.0‐3.3)**	131	0	0.3	0	2.7 (1.5‐4.1)
		70–74		11	3.7	**3.0 (1.5**–**5)**			4	0.9	**4.5 (1.2**–**10)**	
		75–79		8	3.6	2.2 (0.9–4)			2	1.2	1.7 (0.2–4.9)	
		80–84		8	4.5	1.8 (0.8–3.3)			3	1.5	2 (0.4–4.8)	
	M	65–69	127	5	1.1	**4.7 (1.5**–**9.8)**		90	0	0.2	0	
		70–74		11	3.0	**3.6 (1.8**–**6.1)**			2	0.6	3.4 (0.3–9.8)	
		75–79		7	3.2	2.2 (0.9–4.1)			2	0.8	2.6 (0.2–7.3)	
		80–84		5	3.2	1.6 (0.5–3.3)			3	0.5	**5.8 (1.1**–**14.3)**	
Schizophrenia	F	65–69	117	0	0.4	0	**3.5 (2.6‐4.5)**	82	0	0.1	0	2.9 (1.5‐4.7)
		70–74		7	1.5	**4.6 (1.8**–**8.7)**			3	0.5	**6.7 (1.3**–**16.3)**	
		75–79		7	2.2	**3.1 (1.2**–**5.9)**			0	0.7	0	
		80–84		10	3.7	**2.7 (1.3**–**4.7)**			6	1.6	**3.8 (1.4**–**7.4)**	
	M	65–69	96	2	0.8	2.4 (0.2–6.8)		71	0	0.2	0	
		70–74		13	3.0	**4.3 (2.3**–**7)**			2	0.7	2.9 (0.3–8.3)	
		75–79		7	1.9	**3.7 (1.4**–**6.9)**			1	0.5	2.1 (0–8.2)	
		80–84		5	1.1	**4.4 (1.4**–**9.1)**			1	0.3	3.5 (0–13.8)	
Recurrent depression	F	65–69	383	4	1.4	2.8 (0.7–6.3)	**2.5 (2.0‐3.0)**	251	0	0.3	0	3.8 (2.6‐5.2)
		70–74		14	4.7	**3.0 (1.6**–**4.7)**			0	0.9	0	
		75–79		17	7.2	**2.4 (1.4**–**3.6)**			6	2.2	2.8 (1–5.4)	
		80–84		22	10.3	**2.1 (1.3**–**3.1)**			10	3.1	**3.2 (1.5**–**5.6)**	
	M	65–69	196	5	1.4	**3.6 (1.1**–**7.4)**		119	2	0.2	8.4 (0.8–24)	
		70–74		10	4.3	**2.3 (1.1**–**4)**			5	0.6	**8.1 (2.5**–**16.7)**	
		75–79		14	4.7	**3.0 (1.6**–**4.8)**			5	0.8	**6.1 (1.9**–**12.6)**	
		80–84		13	5.6	**2.3 (1.2**–**3.8)**			5	0.5	**9.8 (3.1**–**20.3)**	
Anxiety	F	65–69	491	4	1.4	2.8 (0.7–6.3)	**1.8 (1.4‐2.2)**	294	2	0.3	7 (0.7–19.9)	4.3 (3.0‐5.7)
		70–74		14	5.5	**2.5 (1.4**–**4)**			3	0.7	4 (0.8–9.8)	
		75–79		14	9.0	1.6 (0.8–2.5)			13	1.6	**8 (4.3**–**13)**	
		80–84		19	15.1	1.3 (0.8–1.9)			10	4.2	**2.4 (1.1**–**4.1)**	
	M	65–69	238	3	0.9	3.4 (0.6–8.3)		136	1	0.1	7.5 (0–29.3)	
		70–74		12	4.3	**2.8 (1.4**–**4.6)**			2	0.5	3.8 (0.4–11)	
		75–79		9	5.2	1.7 (0.8–3.1)			5	0.6	**8.2 (2.6**–**17)**	
		80–84		14	8.4	1.7 (0.9–2.7)			3	1.0	2.9 (0.5–7)	

*Notes*: SMR and SIR shown, by gender (F and M) and age of follow‐up, and shown overall for the SMI (statistically significant in bold). N = number of patients contributing to SMI and gender group.

Abbreviations: CI, confidence interval; F, female; M, male; SIR, standard incidence ratios; SMI, serious mental illness; SMR, standard mortality ratio.

### 3.3 Dementia diagnosis

After exclusions, there were 1339 patients for dementia analysis. Within the 1339, there were 20/240 (8.3%) patients in the bipolar/mania group with a dementia diagnosis, 18/167 (10.8%) in the schizophrenia group, 53/436 (12.2%) in the recurrent depression group and 52/496 (10.5%) in the anxiety group.

Competitive risk regression (Table 2) showed no significant difference between the four SMI groups. A significant association of dementia with age (*p* < 0.0001) and referral to the memory clinic (*p* = 0.001) was shown. In the sensitivity analysis, repeating this competing risk analysis in SMI groups which were not mutually exclusive, again no significant difference between the SMI groups was shown (Table S3).

**TABLE 2:** Competing risk regression analysis in 1339 people in four SMI groups, for outcome dementia, in patients with at least a year of follow‐up from index referral

		*N*	Hazard Ratio (95% CI)	*p* value
Age	Years	1339	1.062 (1.042, 1.08)	<0.0001
Sex	Female	886	Ref	‐
	Male	453	1.128 (0.784, 1.62)	0.52
SMI	Anxiety	496	Ref	‐
	Recurrent depression	436	1.027 (0.691, 1.53)	0.89
	Schizophrenia	167	0.828 (0.468, 1.47)	0.52
	Bipolar/mania	240	0.661 (0.385, 1.13)	0.13
Treatment setting	Others	820	Ref	‐
	Memory clinic	117	2.388 (1.420, 4.02)	0.001
	Crisis resolution team/home treatment	208	1.120 (0.697, 1.80)	0.64
	Liaison psychiatry	194	1.136 (0.698, 1.85)	0.61

*Note*: The risk of developing dementia did not vary significantly with SMI.

Abbreviation: CI, confidence interval; SMI, serious mental illness.

SIRs, observed dementia diagnoses as a ratio to expected in the general population given the same follow‐up time, were greater than one in 25/32 subgroups, although most comparisons did not reach statistical significance (Table 1). SIR point estimates were particularly high in males with recurrent depression. Males aged 80–84 with recurrent depression showed the highest point estimate for SIR (9.8 [95% CI 3.1–20.3]). The SIR for anxiety (all eight subgroups combined) was the highest of the four SMI groups (4.3 [95% CI 3.0–5.7]), and for bipolar/mania the lowest (2.7 [95% CI 1.5–4.1]).

## 4 DISCUSSION

The rates of death and dementia in this older population referred into secondary care psychiatric services with SMI were higher than general population rates. Patients with schizophrenia showed the highest hazard rate for death compared to other SMI groups. In comparison of treatment settings, mortality rates were highest in the liaison psychiatry group. Compared with the general population, patients with SMIs showed higher mortality rates, especially in the younger age categories. Although the cause of death is not known, the fact that patients referred into liaison psychiatry had a particularly high mortality rate, suggests that deaths tend to be due to physical causes and underlines the high mortality risk associated with referral into hospital with conditions such as delirium as opposed to patients well enough to attend community clinics.

The risk of dementia did not vary significantly by the preceding SMI, although anxiety and recurrent depression showed higher point estimates than schizophrenia and bipolar disorder. We note that numbers in the comparisons with the general populations were small particularly in age and gender subgroups and comparisons with the general population not always significant. Previous studies have highlighted dementia rates that are increased in bipolar disorder,^6^ ^,^ ^18^ ^,^ ^19^ schizophrenia,^20^ and depression^18^ ^,^ ^19^ ^,^ ^21^ compared with a population control. This was the case in this study too and adds to this literature in suggesting that dementia rates are raised across a wide range of psychiatric presentations compared with the general population, including anxiety disorders in the context of secondary care services.

A significantly increased burden of cardiovascular risk factors has been found in bipolar disorder, which been suggested as a possible explanation for the increased risk of death and dementia in bipolar disorder.^9^ ^,^ ^22^ Other mechanisms have been suggested to explain the link between depression and dementia, including that depression reduces cognitive reserve through glucocorticoid effects on the brain.^18^ The distinction between early and late‐onset depression may be helpful as ‘late onset’ depression may be a prodrome to dementia^21^ ^,^ ^23^ although ‘early onset’ cannot be excluded in a recurrent depression presenting again in later life. Diagnosing dementia in older people with SMIs is complex and difficult as the reduced ability to perform activities of daily living may initially be attributed to the SMI rather than a degenerative process. This may lead to under‐recognition of dementia in patients with severe SMIs. The relatively high risk of dementia in this study, particularly in the anxiety group and recurrent depression groups highlights but does not prove that these may be prodromal symptoms of dementia. Detailed life‐course data enabling differentiation between longstanding versus recent‐onset disorder, were not available to us in this study. Further research to determine whether SMIs provide a causal link to dementia is required and this has not specifically been addressed in our study.

Rates of death and dementia measured in this study are generally high compared to previous studies, particularly the risk of dementia in recurrent depression and anxiety. In a previous cohort study of older men, bipolar disorder was associated with an increased age‐adjusted mortality hazard ratio (1.51, 95% CI 1.28–1.77) and increased adjusted HR of dementia (2.30, 95% CI 1.80–2.94) compared to those without bipolar.^6^ In a population based study of depression followed up for 6 years for death, the unadjusted relative risk was 1.41 (95% CI 1.22–1.63).^7^ In a large community‐based study, late‐life depression was associated with an increased hazard rate for dementia (1.46, 95% CI 1.16, 1.84).^21^ The high risks relative to the general population in our study could be accounted for by our context of referrals into secondary care; a more severely ill population than those who remain in primary care, care by mental health specialists trained to identify dementia and in the case of depression and anxiety and risk of dementia a high proportion presenting with prodromal dementia as well as methodological differences.

Using a service secondary care database presented challenges. Since patients are often referred into secondary care due to the onset of symptoms, a high load of dementia cases at referral is expected (prevalent cases). As in the classical cohort study design, to compare between the four SMI groups, prevalent cases are removed, and new or incident cases identified. However, it takes time from referral for the process of clinical assessment and entry of the dementia diagnosis. We removed prevalent cases by setting a cut‐off point allowing a certain period of time for the process of dementia diagnoses to be made. Results could vary depending on the cut‐off point chosen. We examined the histogram of dementia cases to choose this point, allowing a year after referral for prevalent dementia cases to be entered, excluding patients with less than a year of follow‐up from referral.

A strength of this study is the inclusion of multiple SMI groups allowing comparison between them. Additionally, we were able to analyse the effect of service process factors on the measured death and dementia rates. Database studies often take data from sources that have had input from secondary care data such as hospital episode data. In our study, since this is data derived directly from our own service provision, we were able to include and examine variables such as treatment setting, which was found to influence the measured mortality rates.

### 4.1 Limitations

A limitation of this study is that we did not have our own control group without SMI. However, we compared our rates to the UK population figures^14^ (17) as an external control. Fortunately, the Cognitive Function and Ageing Studies II,^17^ which provided the UK population dementia incidence rates (per 1000 person years), included Cambridgeshire as one of the areas in rate calculation. However, our study group was a referred population, perhaps with increased risk of dementia diagnosis due to repeated reviews by clinicians that the general population is not exposed to (surveillance bias). Measurement of rates of mortality and dementia in a community setting with and without psychiatric disorders is needed to confirm these results.

Additionally, we used clinician entered ICD‐10 diagnoses to identify the patients with SMI and the patients with dementia, without further validation for the study. However, diagnoses can be cancelled by clinicians if considered incorrect and cancelled diagnoses were not included in the study. A significant limitation of this study is that not all patients have a diagnosis code entered implying missing diagnoses or lack of capture of all true diagnoses. Those uncoded may differ from the coded group in some undetermined way, introducing a possible bias. Furthermore, we expect to have missed some dementia cases within our SMI groups, because not all correct diagnoses are entered by the clinician. The number of missed cases is unquantified, but this would tend to lead to an underestimate of the rate of dementia in the study population.

Also, although we removed cases of dementia prevalent at referral by delaying the start of follow‐up until at least a year after referral, some prevalent cases are likely included erroneously as incident cases when the dementia diagnosis was entered later.

Finally, cause of death and comorbidity variables would be useful to investigate underlying reasons for increased mortality in groups such as those referred to liaison psychiatry. These variables were not reliably available in the CPFT research database at the time of this study but will likely provide a useful direction for future research as they become available.

### 4.2 Conclusions

This study points to high rates of mortality and of dementia in a psychiatric secondary care elderly population across a range of SMI groups, including patients who present to services with anxiety. In the case of dementia, further research is needed to differentiate whether psychiatric disorders across the range act as risk factors or rather as prodrome heralding dementia onset or both. Further research to examine the cause of death and reasons for increased dementia diagnosis in this population is warranted. Continued efforts need to be made to reduce risks for dementia and higher mortality rates in both young and older people with mental health disorders. Having data on mortality established in this secondary care population will provide a helpful baseline for comparison with future mortality to evaluate service changes designed to decrease mortality in this vulnerable group.

## CONFLICT OF INTEREST

The authors declare that there is no conflict of interest. RNC consults for Campden Instruments Ltd and receives royalties from Cambridge University Press, Cambridge Enterprise, and Routledge.

## AUTHOR CONTRIBUTIONS

Anne Kershenbaum, Aida Seyedsalehi, Benjamin R Underwood, Judy Sasha Rubinsztein, Rudolf N Cardinal and Shanquan Chen participated in the design of the study. Anne Kershenbaum and Jonathan Lewis were responsible for the data acquisition. Anne Kershenbaum, Rudolf N Cardinal and Shanquan Chen performed the data analysis and interpretation. Anne Kershenbaumand Judy Sasha Rubinsztein drafted the manuscript. All authors critically evaluated the manuscript and gave their final approval before submission.

## Supporting information

Supporting Information 1

## Data Availability

Patient‐level data is not publicly available, under NHS Research Ethics terms. Source code and summary data are available on request.
