# Supplementary material for: Investigation of risk of dementia diagnosis and death in patients in older people's secondary care mental health services
Source: Int J Geriatr Psychiatry. 2020 Nov 4;36(4):573–82. doi: 10.1002/gps.5455 (PMC7984055; doi:10.1002/gps.5455)
Supplement: Supplementary file 1 — Supporting Information 1 [file GPS-36-573-s001.docx]

**Supplementary Figure 1. Table linkages by research ID (RID).**

REFERRALS TABLE (includes RID, date of referral, date of discharge, service referred to) 374,589 rows and 138,462 people, after exclusion referrals before 2013, 336,257 rows and 126,235 people

After exclusion of all referrals before 2013, 303,244 rows, 118,550 people

28,344 people age 65 and over at first recorded referral, 4 excluded in data cleaning leaving **28,340 people in base population**

Includes

401 patients with bipolar/mania (F30*/31*) code

290 patients with schizophrenia (F20*) code

849 patients with recurrent depression (F33*) code

1152 patients with anxiety (F40*-41*) code

Due to some patients having codes that fall into multiple groups, four non-overlapping groups were created using the following hierarchy bipolar/mania>schizophrenia>recurrent depression>anxiety creating the study population

Group 1) 401 patients with bipolar/mania (F30*/31*) code; 356 with > year of follow-up for death and 253 for dementia (355 for mortality after removal death before or same day as bipolar/mania diagnosis and 240 for dementia after removal dementia diagnoses before or same day as bipolar/mania diagnosis)

Group 2) 284 patients with schizophrenia (F20*) code (not in group 1); 238 with > year of follow-up for death and 172 for dementia (167 for dementia after removal dementia diagnoses before or same day as schizophrenia diagnosis)

Group 3) 824 patients with recurrent depression (F33*) code (not in group 1/2); 715 with > year of follow-up for death and 461 for dementia (436 for dementia after removal dementia diagnoses before or same day as recurrent depression diagnosis)

Group 4) 1063 patients with anxiety (F40*-41*) code (not in group 1/2/3); 877 with > year of follow-up for death and 517 for dementia (496 for dementia after removal dementia diagnoses before or same day as anxiety diagnosis)

32,666 have a diagnosis recorded

221160160752250


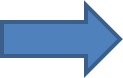


DIAGNOSIS TABLE (includes RID, diagnosis code)

74,750 rows and 36,989 people

Removal of duplicates leaving 74,228 rows and 36,989 people

Removal of cancelled diagnoses leaving 69,740 rows and 36,884 people

17,662 people of the 28,340 base population had a diagnosis assigned including

28,089 rows; F00-F03, Dementia, 12004; F04-F09, 6090; F10-F19, 470; F20, Schizophrenia,443; F21-F29, 366; F30-F31, Bipolar/mania, 679; F32-F39 (excluding F33),2513; F33, Recurrent depression, 1093; F40-F41, Anxiety, 1330; F42-F49, 645; F50-F59, 35; F60-F69, 181; F70-F98, 54; F99, 148; Non-F codes, 2038

DEMOGRAPHY TABLE (RID, date of birth, date of death, sex)

198,045 rows and people

| Year of referral | no diagnosis code | any diagnosis code | percentage with code % |
| --- | --- | --- | --- |
| 2013 | 2548 | 3124 | 55.1 |
| 2014 | 1549 | 2850 | 64.8 |
| 2015 | 1329 | 2992 | 69.2 |
| 2016 | 1170 | 2870 | 71.0 |
| 2017 | 1266 | 2482 | 66.2 |
| 2018 | 1453 | 2372 | 62.0 |
| 2019 | 1363 | 972 | 41.6 |

**Supplementary Table 1. Percentage of patients with a code entered by year of referral in base** **population (n=28,340) (In 2019 referrals in records till 19/8/19).**

|  |  | Anxiety  N=1063 | Recurrent  depression  N=824 | Schizophrenia  N=284 | Bipolar/mania  N=401 |
| --- | --- | --- | --- | --- | --- |
| Age group at referral | 65-74 | 484(45.5%) | 398(48.3%) | 178(62.7%) | 250(62.3%) |
|  | 75-84 | 413(38.9%) | 300(36.4%) | 78(27.5%) | 129(32.2%) |
|  | 85+ | 166(15.6%) | 126(15.3%) | 28(9.9%) | 22(5.5%) |
|  |  |  |  |  |  |
| Sex | Female | 741(69.7%) | 542(65.8%) | 164(57.7%) | 241(60.1%) |
|  | Male | 322(30.3%) | 282(34.2%) | 120(42.3%) | 160(39.9%) |
|  |  |  |  |  |  |
| Treatment setting | Liaison | 218(20.5%) | 204(24.8%) | 72(25.4%) | 102(25.4%) |
|  | Memory Services | 136(12.8%) | 80(9.7%) | 16(5.6%) | 25(6.2%) |
|  | Crisis/Home treatment | 94(8.8%) | 132(16.0%) | 56(19.7%) | 80(20.0%) |
|  | Others | 615(57.9%) | 408(49.5%) | 140(49.3%) | 194(48.4%) |

**Supplementary Table 2. Descriptive statistics for study population, four diagnosis groups, N=2,572.**

**Supplementary Figure 2a. Survival, by serious mental illness (SMI) and initial treatment setting, in patients with at least a year of follow-up (2,185 people) – Cox sensitivity analysis (A) controlling for age, gender, and treatment setting as before, but now also controlling for dementia.**


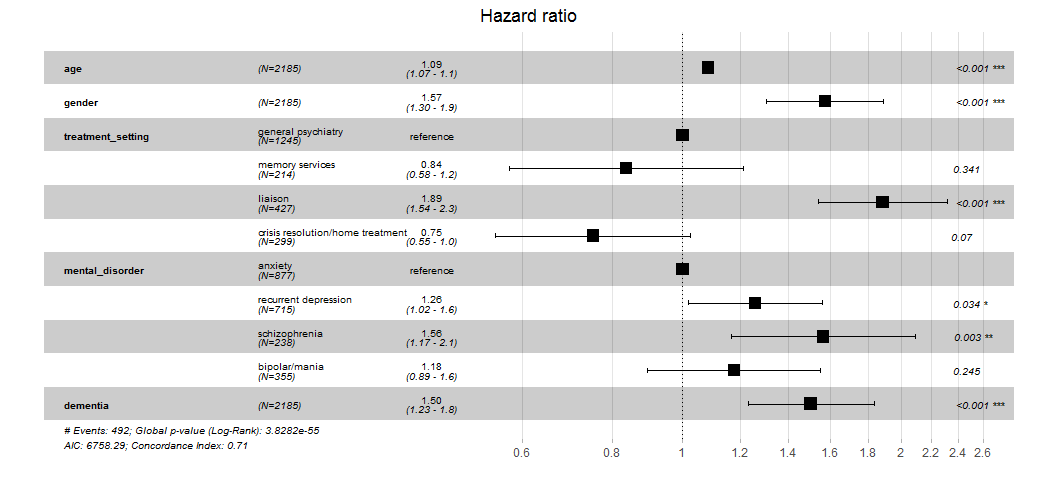


**Supplementary Figure 2b. Survival, by serious mental illness (SMI) and initial treatment setting, in patients with at least a year of follow-up – Cox sensitivity analysis (B), excluding the 371 patients with dementia, leaving 1,814 people.**


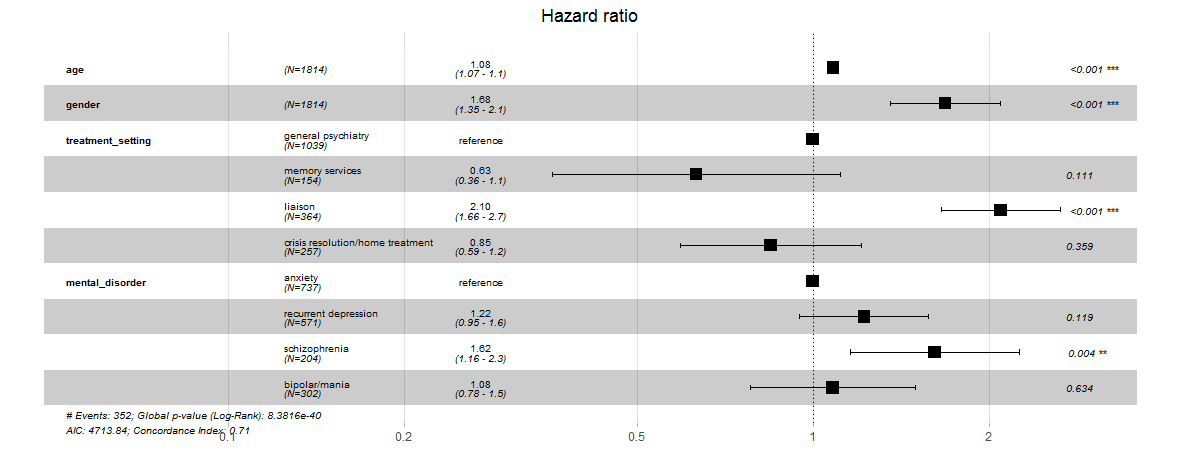


|  |  | Mortality analysis | | | |  | Dementia analysis | | | |
| --- | --- | --- | --- | --- | --- | --- | --- | --- | --- | --- |
|  |  | N | deaths | % | Hazard Ratio (95% CI) |  | N | dementia | % | Hazard Ratio (95% CI) |
| SMI | Anxiety | 877 | 163 | 18.6 | Ref |  | 496 | 52 | 10.5 | Ref |
|  | Recurrent depression | 641 | 171 | 26.7 | 1.3(1.1-1.6) |  | 376 | 46 | 12.2 | 1.0(0.7-1.6) |
|  | Schizophrenia | 234 | 63 | 26.9 | 1.5(1.1-2.1) |  | 164 | 17 | 10.4 | 0.8(0.4-1.4) |
|  | Bipolar/mania | 318 | 70 | 22.0 | 1.2(0.9-1.6) |  | 209 | 17 | 8.1 | 0.7(0.4-1.2) |
|  | Anxiety plus Recurrent depression | 74 | 13 | 17.6 | 1.0(0.6-1.8) |  | 60 | 7 | 11.7 | 1.0(0.4-2.1) |
|  | Anxiety plus Schizophrenia | 1 | 0 | 0.0 |  |  | 1 | 0 | 0.0 |  |
|  | Anxiety plus Bipolar/mania | 10 | 2 | 20.0 | 1.1(0.3-4.3) |  | 9 | 1 | 11.1 | 1.0(0.1-7.2) |
|  | Recurrent depression plus Schizophrenia | 3 | 2 | 66.7 | 11.7(2.8-48.5) |  | 2 | 1 | 50.0 | 2.5(0.7-9.3) |
|  | Recurrent depression plus Bipolar/mania | 22 | 5 | 22.7 | 0.9(0.4-2.3) |  | 18 | 2 | 11.1 | 0.8(0.2-3.0) |
|  | Bipolar/mania plus Schizophrenia | 5 | 3 | 60.0 | 2.6(0.8-8.3) |  | 4 | 0 | 0.0 |  |

**Supplementary Table 3. Hazard ratio with 95% confidence interval for mortality and dementia incidence for SMI** **(serious mental illness) where SMIs not mutually exclusive).**

|  |  |  | Deaths | | | | |
| --- | --- | --- | --- | --- | --- | --- | --- |
| SMI |  | Age | N | O | E | SMR (95% CI) for subgroup | SMR (95% CI) for disorder |
| Bipolar/mania | F | 65–69 | 165 | 4 | 0.9 | **4.5(1.2-10.1)** | **2.5(1.8-3.2)** |
|  |  | 70–74 |  | 10 | 3.4 | **2.9(1.4-5.1)** |  |
|  |  | 75–79 |  | 6 | 2.9 | 2.0(0.7-4.0) |  |
|  |  | 80–84 |  | 3 | 3.0 | 1.0(0.2-2.4) |  |
|  | M | 65–69 | 113 | 4 | 0.9 | **4.4(1.1-9.7)** |  |
|  |  | 70–74 |  | 9 | 2.8 | **3.2(1.5-5.7)** |  |
|  |  | 75–79 |  | 7 | 2.8 | 2.5(1.0-4.7) |  |
|  |  | 80–84 |  | 4 | 2.3 | 1.7(0.4-3.8) |  |
| Schizophrenia | F | 65–69 | 101 | 0 | 0.4 |  | **3.3(2.4-4.4)** |
|  |  | 70–74 |  | 5 | 1.4 | **3.7(1.2-7.6)** |  |
|  |  | 75–79 |  | 6 | 2.1 | 2.9(1.0-5.7) |  |
|  |  | 80–84 |  | 8 | 2.9 | **2.8(1.2-5.1)** |  |
|  | M | 65–69 | 87 | 2 | 0.8 | 2.4(0.2-7.0) |  |
|  |  | 70–74 |  | 9 | 2.5 | **3.5(1.6-6.2)** |  |
|  |  | 75–79 |  | 7 | 1.7 | **4.2(1.7-7.8)** |  |
|  |  | 80–84 |  | 5 | 0.9 | **5.8(1.8-12.0)** |  |
| Recurrent depression | F | 65–69 | 313 | 4 | 1.4 | 2.9(0.8-6.5) | **2.2(1.7-2.7)** |
|  |  | 70–74 |  | 11 | 4.3 | **2.5(1.3-4.3)** |  |
|  |  | 75–79 |  | 11 | 5.8 | 1.9(0.9-3.2) |  |
|  |  | 80–84 |  | 10 | 6.6 | 1.5(0.7-2.6) |  |
|  | M | 65–69 | 165 | 5 | 1.3 | **4.0(1.2-8.2)** |  |
|  |  | 70–74 |  | 9 | 3.6 | **2.5(1.1-4.4)** |  |
|  |  | 75–79 |  | 11 | 3.8 | **2.9(1.5-4.9)** |  |
|  |  | 80–84 |  | 6 | 4.0 | 1.5(0.5-3.0) |  |
| Anxiety | F | 65–69 | 425 | 4 | 1.3 | 3.0(0.8-6.7) | **1.7(1.4-2.2)** |
|  |  | 70–74 |  | 12 | 5.3 | **2.3(1.2-3.7)** |  |
|  |  | 75–79 |  | 13 | 7.2 | 1.8(1.0-2.9) |  |
|  |  | 80–84 |  | 11 | 12.1 | 0.9(0.4-1.5) |  |
|  | M | 65–69 | 205 | 2 | 0.8 | 2.4(0.2-6.9) |  |
|  |  | 70–74 |  | 10 | 4.0 | **2.5(1.2-4.3)** |  |
|  |  | 75–79 |  | 9 | 4.1 | 2.2(1.0-3.9) |  |
|  |  | 80–84 |  | 11 | 6.5 | 1.7(0.8-2.8) |  |

**Supplementary Table 4. Observed deaths in study (O) and expected in general population given same follow-up time (E) in patients with at least a year of follow-up from referral and without patients with dementia. Standard mortality ratio (SMR) shown, by gender (female = F, male =M) and age of follow-up, and shown overall for the serious mental illness (SMI) (statistically significant in bold). N=number of patients contributing to SMI and gender group.**
